# Society Meetings

**Published:** 1918-08

**Authors:** 


### SOCIETY MEETINGS

---

Brief reports and announcements of meetings of societies of Western New York, and those of general scope, are requested from Secretaries. Copy should be on hand the fifteenth of the month. Full transactions will be published at cost of composition.

---

**The Chemung Co. Medical Society** has increased its professional fees, making house visits $2.00; night visits $4.00 and maternity cases, minimum fee $25.00.

---

**Buffalo Academy of Medicine,** Meeting June 28th. Subject: "The Physical Training of the Public School Children."

---

**The Lake Keuka Medical and Surgical Assn.** held its 19th annual meeting at Gibson's Landing, Lake Keuka, July 26-7. Drs. Howard L. Prince, Rochester; M. M. Lucid, Cortland; Walter B. Weidler, New York; M. P. Messinger, Oakfield; Robert Knight, Seneca Falls; A. McKenzie, Montreal; T. F. Laurie, Auburn; A. H. Paine, Rochester; Harvey Jack, Hornell; W. J. Johnson, Batavia, and Ledra Heazlit, Auburn, read papers.

---

The twenty-eighth annual session of the **New York and New England Association of Railway Surgeons** will be held at the Hotel McAlpine, New York City, on Oct. 21st. A symposium will be presented on the "Modern Treatment of Infected Wounds." Dr. J. S. Hill, President, Bellows Falls, Vermont; Geo. Chaffee, Cor. Sec., Little Meadows, Pa.

---

**The American Gastro-Enterologic Assn.** has elected the following officers: Pres. Walter A. Bastedo, N. Y. City; Vice-Pres. Thos R. Brown, Baltimore, and Franklin W. White, Boston; Sec.-Treas. Frank Smithies, Chicago; Recorder, Horace W. Soper, St. Louis.

